# Fast Chromatographic Determination of Free Amino Acids in Bee Pollen

**DOI:** 10.3390/foods11244013

**Published:** 2022-12-12

**Authors:** Beatriz Martín-Gómez, Laura Salahange, Jesús A. Tapia, María T. Martín, Ana M. Ares, José Bernal

**Affiliations:** 1 Analytical Chemistry Group (TESEA), I. U. CINQUIMA, Faculty of Sciences, University of Valladolid, 47011 Valladolid, Spain; 2 Department of Statistics and Operations Research, Faculty of Sciences, University of Valladolid, 47011 Valladolid, Spain

**Keywords:** authentication, bee pollen, bioactive compounds, food analysis, food quality, free amino acids, GC-MS, LC-MS, markers, principal component analysis

## Abstract

The consumption of bee pollen has increased in the last few years due to its nutritional and health-promoting properties, which are directly related to its bioactive constituents, such as amino acids. Currently, there is great interest in understanding the role of these in bee products as it provides relevant information, e.g., regarding nutritional value or geographical and botanical origins. In the present study, two fast chromatographic methods were adapted based on commercial EZ:faast™ kits for gas chromatography-mass spectrometry and liquid chromatography–mass spectrometry for determining free amino acids in bee pollen. Both methods involved the extraction of amino acids with water, followed by a solid phase extraction to eliminate interfering compounds, and a derivatization of the amino acids prior to their chromatographic separation. The best results in terms of run time (<7 min), matrix effect, and limits of quantification (3–75 mg/kg) were obtained when gas chromatography–mass spectrometry was employed. This latter methodology was applied to analyze several bee pollen samples obtained from local markets and experimental apiaries. The findings obtained from a statistical examination based on principal component analysis showed that bee pollen samples from commercial or experimental apiaries were different in their amino acid composition.

## 1. Introduction

Bee products, such as honey, royal jelly, or bee pollen, have been consumed since ancient times for their nutritional value and health promoting effects (of an antioxidant, anti-inflammatory, anti-cancer, analgesic, anti-fungal or anti-viral nature) [1,2,3,4]. The consumption of bee products has been increasing in interest in the last few years, and this is particularly relevant in the case of bee pollen [5,6]. However, the production of bee products cannot undergo a rapid growth in the short term, and this may result in fraudulent practice in the form of adulteration [7], which is causing significant damage to the beekeeping industry. Therefore, the authentication of bee products, especially honey and bee pollen, in terms of botanical and geographical origins is essential to protect consumer health and to avoid fraudulence [8]. One of the strategies employed to authenticate the origin of bee pollen is the study of its composition, as it is well-known that it is mainly dependent on the type of plant and the geographical origin [5,9,10]. Consequently, in the last few years, different families of compounds (lipids, phenolic compounds, betaines, glucosinolates, minerals and amino acids) have been examined in bee pollen not only to determine their nutritional or bioactive properties, but also regarding their function as markers of its origin [11,12,13,14,15,16,17].

Amino acids are responsible for a large part of the biological activity of bee pollen. They play an important role in human nutrition (e.g., in metabolism, reducing excessive body fat) [18]. They have been extensively studied in bee pollen in the last few years [5,9,17,18,19,20,21,22], with the primary objective of characterizing bee pollen as regards its botanical or geographical origin, or to evaluate its nutritional value. It should be mentioned that we have recently demonstrated the potential of amino acids as markers of the apiary of origin and harvesting period [5], which represents a significant advance in the authentication of this product. However, the overall analysis time per sample, including sample treatment (solvent extraction and on-line derivatization) and chromatographic analysis (HPLC with fluorescence detector), is very high (>1 h), which could affect its applicability for analyzing many samples. The relatively long period of time required for both sample preparation and analysis is a common problem when determining amino acids by chromatographic techniques [23]. Fortunately, this procedure could be expedited by using some commercial kits (EZ:faast^TM^, Phenomenex, Torrance, CA, USA). Simple solid-phase extraction (SPE) and rapid derivatization of the amino acids combine to shorten preparation time considerably, and analysis time varies between 7 (gas chromatography-mass spectrometry, GC-MS) and 17 min (high performance liquid chromatography-mass spectrometry, HPLC-MS). These kits have previously been used in different food matrices [23], including honey [24], but to our knowledge they have never been used in bee pollen.

Thus, the aim of the present study was to evaluate for the first time the potential of the EZ:faast^TM^ GC-MS and HPLC-MS kits for determining free amino acid analysis in bee pollen. Additionally, the analytical performances of both methods were compared to choose the best option in terms of overall analysis time, sensitivity (limits of quantification, LOQ), matrix effect, and precision. Further aims of this work concerned determining the free amino acid content in bee pollen samples from different origins (commercial and experimental apiaries), and comparing these by means of chemometrics, or, more specifically, principal component analysis (PCA).

## 2. Materials and Methods

### 2.1. Chemical and Materials

Solutions with mixing standards prepared from analytical grade standards at a concentration of 200 nmol/mL (see Table 1 and Table 2), reagents and organic solvents (see Section 2.3.2) were supplied in the EZ:faast^TM^ GC-MS and LC-MS kits for free amino acid analysis (Phenomenex, Torrance, CA, USA). Ammonium formate (purity ≥ 97%) was supplied by Sigma-Aldrich Chemie Gbmh (Steinheim, Germany), while methanol (HPLC-grade) was obtained from LabScan Ltd. (Dublin, Ireland). Syringe filters (17 mm, Nylon 0.45 μm) were purchased from Nalgene (Rochester, NY, USA), and ultrapure water was obtained from Millipore Milli-RO plus and Milli-Q systems (Bedford, MA, USA). An Eppendorf Centrifuge 5810R (Hamburg, Germany), a Moulinette chopper device from Moulinex (Paris, France), as well as a Vibromatic mechanical shaker, a Vortex device, and a drying oven from J.P. Selecta S.A. (Barcelona, Spain) were used for the sample treatment.

### 2.2. Standards

Standard in solvent solutions were prepared as indicated in the corresponding GC-MS and HPLC-MS EZ:faast^TM^ kits. Briefly, different volumes of the amino acid mixtures supplied in the kits (200 nmol/mL) were mixed with the internal standard solution to obtain five different calibration levels (LOQ (see Table 1 and Table 2), 20, 50, 100, and 200 nmol/mL). It should be mentioned that the standard mixtures of amino acid standards were prepared following the sample treatment described in Section 2.3.2, and that the concentration of each internal standard (IS; homoarginine (HARG) and methionine-*d3* (MET-*d3*), HPLC-MS; norvaline (NVAL), GC-MS) should be of 200 nmol/mL). On the other hand, matrix-matched standards were prepared to evaluate the analytical performance of the method (see Section 3.1.2). The only difference in relation to the standard in solvent solutions was the use of bee pollen samples (0.05 g (HPLC-MS) or 0.10 g (GC-MS)) which were spiked after the extraction with ultrapure water with different volumes of the free amino acid standards (LOQ-200 nmol/mL) and the internal standards at the same concentration (200 nmol/mL).

It should be noted that all the bee pollen samples analyzed contained endogenous free amino acids. Thus, to calculate the signal for the spiked bee pollen samples, the areas corresponding to endogenous levels had to be determined. These areas were subtracted from the total area obtained for the spiked samples. Stock amino acid solutions provided in the kits should be placed in the freezer after use. Meanwhile, calibration solutions were stored in glass containers in darkness at +4 °C. All solutions remained stable for over 2 weeks.

### 2.3. Sample Procurement and Treatment

#### 2.3.1. Samples 

Bee pollen samples were obtained from four apiaries with homogeneous colonies of *Apis mellifera iberiensis* (one representative sample per apiary, *n* = 4; A1–A4) and from local markets in Valladolid (Spain; *n* = 8; C1–C8). It must be remarked that all the commercial samples were labelled as multifloral, and the specific geographical origin was not provided, as it was only stated that were produced in Spain. Moreover, it should be mentioned that a representative sample from each apiary (Pistacho, Fuentelahiguera, Tío Natalio, and Monte), located on the province of Guadalajara (Spain; see Supplementary Materials, Figure S1), was analyzed according to the results summarized in our recent study [5]. 

In addition, bee pollen samples were collected using pollen traps placed at the entrance of the hive. Every two weeks, the pollen trap grid was closed for a period of 24 h in the different hives. In the present study, samples were collected in June (2018). The pollen stored in the collection drawer during this period was collected, immediately sealed, identified, and taken to the laboratory where it was frozen until analysis.

#### 2.3.2. Sample Treatment

**Figure 1:**
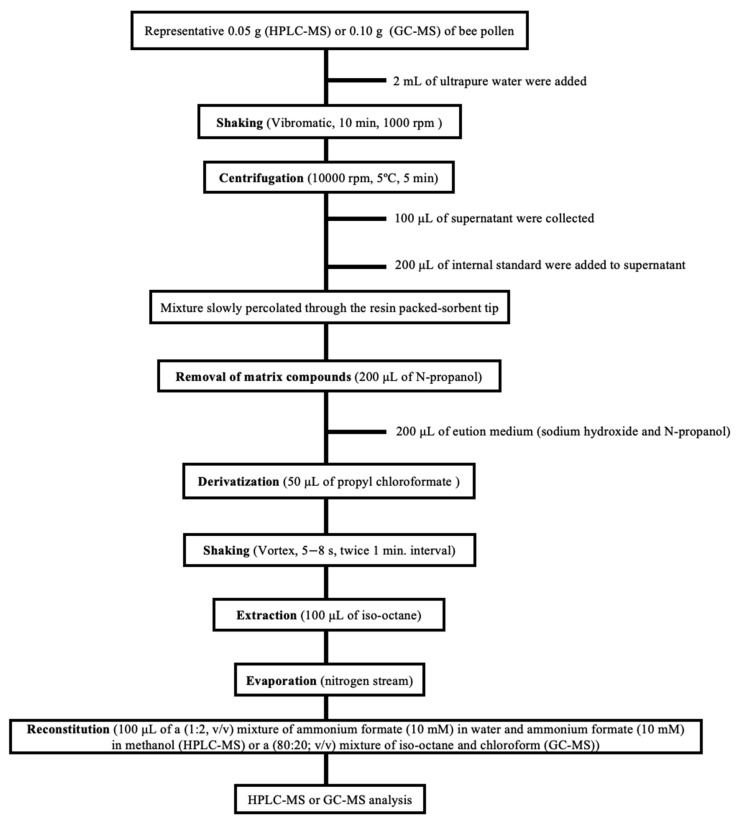
Scheme of the proposed sample treatment.

Bee pollen samples were mixed, ground, and pooled for optimum sample homogeneity. Next, the pollen was dried until the mass stabilized. Subsequently, it was stored in the dark at −20 °C until analysis. Samples were treated according to the procedures described in the EZ:faast^TM^ GC-MS and LC-MS kits, and the only differences were the amount of bee pollen and the solvent used in the final reconstitution step. Figure 1 outlines the steps of the sample treatment study.

### 2.4. Chromatographic Systems

Chromatographic conditions were adapted from those recommended in the EZ:faast™ GC-MS and HPLC-MS kits (Phenomenex) for free amino acid analysis. 

#### 2.4.1. GC-MS Conditions

An Agilent Technologies (Palo Alto, CA, USA) 6890 GC coupled to an Agilent Technologies 5973 MS equipped with an ALS 7863 autosampler and MS ChemStation E 01.00.237 software (Agilent Technologies) was employed. The chromatographic column was a Zebron ZB-AAA (10 m × 0.25 mm × 0.25 μm) from Phenomenex. Separation and detection conditions are summarized in Table 3. It should be mentioned that scan mode (50–450 *m/z*) was used for data acquisition to identify possible compounds characteristic of the locations. Meanwhile, quantification was performed in selected ion monitoring (SIM) mode, with one target/quantification and two qualifier ions for each analyte (see Table 1). 

**Figure 2:**
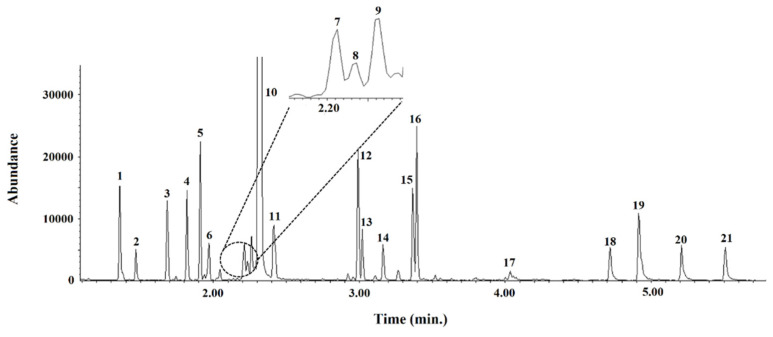
Representative chromatogram (total ion chromatogram (TIC) mode using the quantification ions; see Table 1) obtained from a standard in solvent mixture of free amino acids. The GC-MS conditions are summarized in Section 2.4.1 and Table 1. 1, ALA; 2, GLY; 3, VAL; 4, NVAL (IS); 5, LEU; 6, ILE; 7, THR; 8, GABA; 9, SER; 10, PRO; 11, ASN; 12, ASP; 13, MET; 14, MET-*d3*; 15, GLU; 16, PHE; 17, GLN; 18, LYS; 19, HIS; 20, TYR; 21, TRP.

As can be seen in the total ion chromatogram (TIC; Figure 2), under optimal GC-MS conditions, all compounds eluted in less than 6 min. It should be mentioned that PRO peak is not complete in Figure 2 due to the fact that the chromatogram was amplified in order to show the minor amino acids, not because it was saturated.

#### 2.4.2. HPLC-MS Conditions

**Figure 3:**
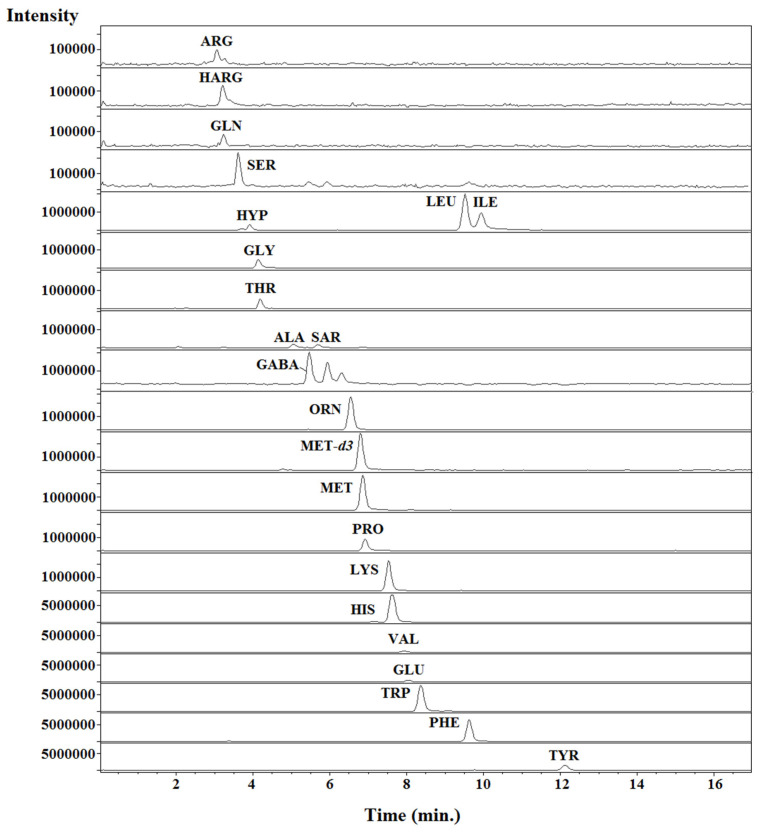
Representative chromatograms (SIM mode using the quantification ions; see Table 2) obtained from a standard in solvent mixture of free amino acids. The HPLC-MS conditions are summarized in Section 2.4.2 and Table 2.

An Agilent Technologies 1100 HPLC coupled to a MS detector (single quadrupole) equipped with an electrospray ionization (ESI) source was selected to perform the analyses. An EZ:faast™ AAA-MS (250 × 3.0 mm, 4 μm; Phenomenex) analytical column was used for separation of the amino acids. Separation and detection conditions are summarized in Table 3. Full-scan spectra were obtained by scanning from *m*/*z* 60 to 600, and quantification was performed in selected ion monitoring (SIM) mode (see Table 2). Under optimal HPLC-MS conditions, all compounds eluted in less than 14 min (see Figure 3).

### 2.5. Statistical Analysis

Statistical analysis was performed by means of SAS PROC PRINCOMP and SAS PROC DISCRIM (version 9.4; SAS Institute Inc., Cary, NC, USA). Firstly, a principal component analysis (PCA) was employed. This is a multivariate technique to summarize data by reducing the number of quantitative variables, and to detect the principal components as linear relationships between the original variables [25]. PCA calculates so many components as quantitative variables have been measured in the sample, and all the PCAs should explain the entire original variability of the data. To determine how many principal components must be used in the discriminant analysis to classify each bee pollen sample in one group, it is important to consider the proportion of accumulated variability explained by the components; if possible, at least 90%. Meanwhile, for a data set containing a classification variable defining groups of observations, the discriminant procedure obtains a criterion to classify each observation into one of the groups. The discriminant function obtained with the PROC DISCRIM program is quadratic when normality is assumed and the homogeneity of covariances is tested.

## 3. Results and Discussion

### 3.1. Chromatographic Methods

#### 3.1.1. Optimization of the Methods

As has already been mentioned in previous sections of this study, the general working conditions of the kits are specified by their manufacturer. Therefore, the conditions selected do not vary largely from those specified in the kits. However, we decided to carry out tests to verify the influence of certain parameters when determining amino acids in bee pollen samples, since, logically speaking, the proposed conditions are general and not adapted to a particular matrix. Firstly, the extraction step of the sample treatment was evaluated with the aim of identifying and quantifying the amino acids present in the pollen in the fastest possible way, while using a minimum of pollen and solvent. As chromatographic analysis was faster with GC-MS, the tests were initially performed with this technique. Free amino acids have generally been extracted from bee pollen with ultrapure water and ethanol [26]. Consequently, we tested both solvents. Results showed that twenty amino acids were identified when using ultrapure water, whilst only four of these (alanine, ALA; phenylalanine, PHE; proline, PRO; valine, VAL) were observed when ethanol was employed. The same behavior was observed with HPLC-MS, although the number of amino acids identified (twenty-three) was greater, as arginine (ARG), ornithine (ORN) and sarcosine (SAR) could now be discerned. Thus, ultrapure water was the solvent chosen to continue the experiments. Subsequently, different amounts of sample and solvent were tested (0.05–1.00 g; 2–5 mL). The best results in terms of the number of compounds detected, the proportion of amino acids extracted, the solvent, and the sample were obtained for GC-MS when using 0.10 g of bee pollen and 2 mL of ultrapure water. Under these conditions twenty amino acids were identified, whereas with the more diluted options, histidine (HIS), lysine (LYS), tyrosine (TYR), and tryptophan (TRP), could not be detected. In relation to HPLC-MS analysis, a different sample amount, in this case the lowest (0.05 g), was chosen as the number of free amino acids extracted was the same as with larger amounts, and the proportion extracted was quite similar.

Regarding the chromatographic conditions, optimization studies were carried out only with the injection volume and the ESI parameters for the HPLC-MS method. Meanwhile, the GC-MS conditions were the same than those specified in the kit, since the results were satisfactory when the recommended conditions were employed. An injection volume of 1 μL is indicated in the kit for HPLC-MS, but with this value few amino acids were identified. Therefore, we decided to test larger injection volumes (5 and 10 μL). These volumes provided better results in terms of identification and quantification, whilst the same number of amino acids were detected with these amounts. However, on quantification of the samples, a difference between the two volumes was observed, since the number of saturated peaks corresponding to certain amino acids was not the same. With 10 μL, several saturated peaks were observed, compared with only two, namely HIS and PRO, when 5 μL was used. Nevertheless, as these peaks also appeared saturated when an injection volume of 1 μL was used, we finally decided to work with an injection volume of 5 μL, bearing in mind the need to dilute the samples of bee pollen with ultrapure water (1:10, *v*/*v*) to determine HIS and PRO by HPLC-MS. This implies that samples analyzed by HPLC-MS should be injected twice if the minority amino acids were not observed in the diluted samples. Moreover, the ESI conditions were also examined, as indicated in the kit. Flow injection analyses were conducted for selecting the optimal ESI-MS parameters in the infusion mode (5 μL/min) of standard solutions of three of the free amino acids (glutamine (GLN), PHE and PRO), the best results being obtained with the conditions detailed in Section 2.4.2. An example of the optimization procedure of fragmentor voltage for GLN is shown in Figure S2 (see Supplementary Materials). 

Under the chosen chromatographic conditions (see Section 2.4), all the compounds were eluted in less than 6 min (GC-MS) or 13 min (HPLC-MS; see Figure 1 and Figure 2), with an overall analysis time, including sample treatment and chromatographic analysis, of close to 30 min (GC-MS) or 50 min (HPLC-MS). It should be noted that the number of compounds identified by HPLC-MS (twenty-three) was slightly larger than with GC-MS (twenty). According to the existing literature, these are not only the fastest chromatographic methods for determining amino acids in bee pollen, but also the proposals with the lowest amounts of solvents required.

#### 3.1.2. Analytical Performance of the Methods

Method selectivity was evaluated by injecting a set of extracts of bee pollen samples (*n* = 6) onto the chromatographic systems, the results being compared with those obtained for the individual standards of the amino acids under study. It was observed that the retention times coincided perfectly in all cases and that there was a great similarity between the MS spectra of the amino acids in standard and bee pollen samples (see Supplementary Materials, Figures S3 and S4). The limits of quantification (LOQs) were determined experimentally as ten times the standard deviation of the intercept for the calibration curve (matrix-matched) divided by the slope [27]. As can be seen in Table 1 and Table 2, the values were lower in all cases when GC-MS was employed. In addition, the LOQs obtained were like those reported in previous studies [5,17,26].

Calibration curves were constructed by plotting the signal on the *y*-axis (analyte peak area/internal standard area) against analyte concentration on the *x*-axis, and calibration standards were prepared as described in Section 2.2. The graphs obtained in all the calibration curves were straight lines, with the coefficient of the determination values (R^2^) above 0.99 in all cases. Working range was verified by examining the deviation of back-calculation concentration from actual concentration (<15%). To evaluate whether or not there was a significant matrix effect for each amino acid, the confidence intervals of the slopes were compared on standard in solvent and matrix-matched calibration curves. In the case of overlap, the slopes were significantly the same at a confidence level of 95%, whereby no matrix effect was considered to have been present. The results are summarized in Table 4, where we see eight amino acids with a significant matrix effect when using GC-MS, and thirteen for HPLC-MS. These results were confirmed when calculating the matrix effect with the following equation: 100 × [1 − (standard in solvent-slope/matrix-matched standard-slope)]. Values higher than 20 mean that a significant matrix effect was observed, which depending on the sign provoked a signal suppression (negative) or enhancement (positive). Therefore, standard in solvent calibration curves could be used for measuring amino acids that were not affected by the matrix effect, while matrix-matched standard calibration curves should be used for the other amino acids.

Finally, experiments to evaluate accuracy were performed. It should be mentioned that accuracy is usually studied as two components: precision and trueness [27]. Measures of precision, expressed as relative standard deviation (%RSD), were performed concurrently by repeated analysis of bee pollen samples, either on the same day (*n* = 6; repeatability [27]), or over three consecutive days (*n* = 6; partial reproducibility [27]). The %RSD values obtained for the areas and retention times were lower than or equal to 10% in all cases and are like those values reported for the analysis of amino acids in honey with the GC-MS kit [24]. Trueness was evaluated by the mean recoveries (as a measure of trueness) calculated by comparing the measured concentrations in spiked samples (see Section 2.2) and theoretical concentrations. Mean recoveries ranged from 85% to 105% (%RSD < 15%) in all cases (see results for some amino acids in Supplementary Materials, Table S1), which are comparable to previous works [5,17,24,26].

#### 3.1.3. Comparison of the Methods

Both chromatographic methods allowed the rapid determination of free amino acids in bee pollen samples, although several differences between them were observed (see Table 5). These mainly related to their analytical performance, since in relation to sample treatment the only difference was the amount employed, which was slightly greater for the GC-MS method.

Firstly, it should be noted that the methods differed in the number of amino acids identified. This was larger in the case of the HPLC-MS method, since ARG, ORN, and SAR cannot be identified by GC-MS. Meanwhile, the GC-MS chromatographic run time (7 min) was three times shorter than that of HPLC-MS (21 min). In addition, the number of amino acids that were affected by the matrix effect with GC-MS was lower. This makes it possible to quantify, if considered necessary, a larger number of amino acids using calibration lines prepared with standard in solvent, which is an easier approach. The sensitivity of the method (LOQs) was also better in the case of the GC-MS method, whilst a further dilution of the sample was required when HPLC-MS was used for determining HIS and PRO, which also implied that two analyses should be performed if the minority compounds were not observed in the diluted samples. Therefore, it may be concluded that the overall performance of the GC-MS method was better than with HPLC-MS, which suggests that the former could be considered the best option for the rapid determination of free amino acids in bee pollen.

### 3.2. Analysis of Bee Pollen Samples

#### 3.2.1. Free Amino Acid Content

Free amino acid content was determined by GC-MS, chosen due to its superior performance, in twelve samples of bee pollen, from four apiaries located in Marchamalo (A1–A4) and eight samples purchased in supermarkets, of multifloral origin and from various regions of Spain. All the samples were analyzed in triplicate by using matrix-matched standard calibration curves in order to simplify the quantification procedure. In view of the results obtained for each of the samples (see Table 6 and Table 7), it may be stated that the twenty amino acids were identified in the samples from the experimental apiaries in a variable concentration range (LOQ-4577 mg/kg), while HIS, LEU, and PHE were not detected in some commercial samples. MET and TYR were detected in all the samples, although in several of these their content was below the LOQs. In addition, the free amino acid content in the samples from the experimental apiaries were quite similar to those values reported in our previous study [5].

It should be noted that MET is difficult to detect in bee pollen with traditional methods involving oven-assisted acid hydrolysis [17], but with the proposed method this problem was solved. In addition, the total content of amino acids was generally greater in bee pollen samples from apiaries than from commercial ones, which could be explained by the drying procedure applied to many foods, including bee pollen, prior to commercialization. This treatment can result in a decrease in amino acid content [28,29]. PRO was the predominant amino acid in most of the samples, which concurs with previous publications [5,9,17,18,21]. When differentiating between the commercial samples and those from the apiaries, it is observed that PRO content was greater in the former, in which this was clearly the predominant amino acid. However, in samples from experimental apiaries, despite PRO remaining a major amino acid, so was asparagine (ASN), a point also found in the literature [22]. This was the predominant amino acid in samples A1 and A3. Glycine (GLY), TYR, PHE, and MET, meanwhile, represented minor amino acids detected in the samples analyzed.

In addition to the difference in PRO content in the samples, it is important to highlight the variation in glutamic acid (GLU) content depending on the origin of the samples, with a greater amount being found in samples from experimental apiaries. Since PRO might originate from GLU because of the dehydration that occurs when bee pollen undergoes drying processes [30], as occurs in commercial pollen samples, it is logical for GLU content to have been lower in commercial samples. Nevertheless, bee pollen constituents, like proteins and amino acids, could be affected for the drying procedure, which is not common, that is usually done prior to the commercialization of bee pollen. This could explain the generally higher free amino acid content in the samples from experimental apiaries. Thus, the composition and nutritional quality could be affected not only by their origin (region, weather, and floral source), but also the drying procedure.

#### 3.2.2. Statistical Analysis

As previously mentioned, all the bee pollen samples were injected into the GC-MS in triplicate. In this way, it was possible to find the differences between the origin (commercial and experimental apiaries) by using the response to the quantitative variables (free amino acids) corresponding to the mean values of the three repetitions of each sample that were made and the concentration-based confidence interval. As can be seen, there were many variables, yet with our statistical approach we were able to reduce the dimensions without losing information, making it possible to obtain a feasible graphic representation. Firstly, the weights of the principal components considering the mean values of the three replicates were obtained; these are linear combinations of the quantitative variables. Each original variable can have a different weight (negative or positive), so that the greater the weight, the more importance that variable has in the final principal component. These values are shown in Table S2 (see Supplementary Materials). The first five principal components accounted for over 90% of the variability (see Supplementary Materials, Table S3). 

**Figure 4:**
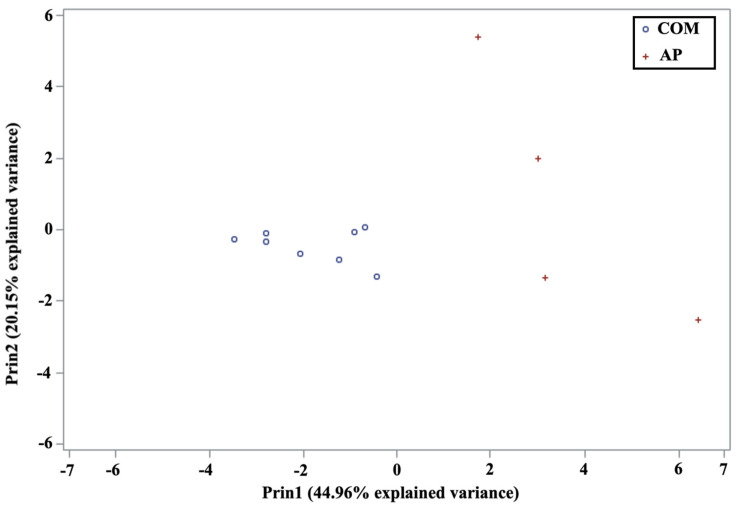
Representation of the bee pollen samples (AP, experimental apiaries; COM, commercial) as function of principal component 1 (Prin1) and 2 (Prin2).

The values of principal components 1 (Prin1) and 2 (Prin2) for the twelve samples were represented (see Figure 4). The first principal component was represented on the *x*-axis. If the values of the variables are represented on the right, it will mean that the positive values will have a greater weight than the negative ones. If, however, they are on the left, it represents, depending on the scale of the graph, a greater weight in values close to zero or negative ones. The second principal component was represented on the *y*-axis. When the values of the variables are very high, the positive values will have the greatest weight, and if the representation of the values is low, the greatest weight will correspond to the negative values of these variables.

As can be seen, commercial samples presented a negative value for the first principal component (see Figure 4), while samples from experimental apiaries have a positive value for this component. Therefore, it has been demonstrated that by considering only the first principal component, it was possible to differentiate the origin (commercial or experimental apiary) of the bee pollen samples analyzed in the present study. In addition, if we examine the data summarized in Table S2, it can be observed that all the amino acids except for PRO and HYP exhibited positive values for the first principal component. This would indicate a greater weight for these amino acids in the commercial samples, grouped on the left side of the graph with negative values. In this study, therefore, these two amino acids could be sufficient to differentiate between the origin (commercial or experimental apiary) of the specific analyzed samples, which concurs with the results presented in previous studies [17]. Moreover, it can also be observed in Figure 4 that the samples obtained from experimental apiaries were not grouped, and differences are clearly visible in their position on the graphic. This result is to be expected considering our recent publication [5], in which we demonstrated that the apiary of origin of the bee pollen samples could be distinguished on the sole basis of their free amino acid content. For that reason, we have only included one representative sample per apiary, as it is enough to show the general trend of those apiaries in relation to the amino acid content.

Finally, we performed a quadratic discriminant analysis with the first two principal components. The results were excellent (see Supplementary Materials, Table S4, as all the samples were correctly assigned to their group of origin (commercial or experimental apiaries). These findings highlighted the potential of free amino acids as bee pollen markers, although, as has been previously mentioned, amino acid content in bee pollen can be also influenced by processing prior to its commercialization. 

## 4. Conclusions

Two fast methodologies have been evaluated for assessing free amino acids in bee pollen samples by means of two different chromatographic techniques, namely GC-MS and HPLC-MS. The methodologies differed in several points. For example, the number of amino acids identified was lower when GC-MS was employed, as ARG, SAR, and ORN could not be identified. Furthermore, the analysis time of the GC-MS method (30 min) was much shorter than with HPLC-MS (50 min), whilst the number of amino acids affected by the matrix effect was lower with GC-MS, and the LOQs values were also better when this was used. Thus, in view of the results obtained, the GC-MS method is proposed as the best option for performing a rapid analysis of amino acids in bee pollen, since, to the best of our knowledge, it is the fastest chromatographic method capable of performing this specific task. Several multifloral bee pollen samples of different origins (commercial or experimental apiaries) were analyzed with the proposed GC-MS method. The results showed that, in general, free amino acid content was greater in the samples from experimental apiaries than from those of commercial sources, with PRO representing the main free amino acid in this case, whilst ASN was also predominant in the samples from experimental apiaries. Moreover, it has been also shown that different bee pollen samples from markets and experimental apiaries presented differences in their amino acid composition. This was corroborated by means of a principal component analysis, and more specifically with the first principal component. The most significant free amino acids in this differentiation were PRO and HYP. In addition, a quadratic discriminant analysis was carried out with the first two principal components. It can be concluded that all the analyzed samples (a 100% success rate) were correctly classified according to their origin (commercial or experimental apiary) on the basis of their free amino acid content. However, it should also be considered that the amino acid composition could be affected not only by the bee pollen origin (region, weather, and floral source), but also its processing, especially drying, prior to commercialization. Finally, these findings confirmed the potential not only of amino acids as biomarkers of pollen origin, but also of the proposed GC-MS method, since it will make it possible to analyze a greater number of samples in less time.

## Figures and Tables

**Table 1:** GC-MS data and limits of quantification for the studied free amino acids.

Amino Acid (Abbreviation)	Retention Time (min)	Ions (*m/z*)	LOQ (mg/kg)
Alanine (ALA)	1.36	130 ^Q,C^, 158 ^C^, 88 ^C^	5
Glycine (GLY)	1.47	116 ^Q,C^, 162 ^C^, 102 ^C^	7
Valine (VAL)	1.69	158 ^Q,C^, 116 ^C^, 72 ^C^	4
Norvaline (NVAL; IS)	1.82	158 ^Q,C^, 116 ^C^, 72 ^C^	NE
Leucine (LEU)	1.92	172 ^Q,C^, 130 ^C^, 86 ^C^	3
Isoleucine (ILE)	1.98	172 ^Q,C^, 130 ^C^, 101 ^C^	5
Threonine (THR)	2.21	101 ^Q,C^, 160 ^C^, 74 ^C^	7
γ-Amino-n-butyric acid (GABA)	2.23	144 ^Q,C^, 172 ^C^, 130 ^C^	18
Serine (SER)	2.26	146 ^Q,C^, 203 ^C^, 60 ^C^	8
Proline (PRO)	2.33	156 ^Q,C^, 243 ^C^, 70 ^C^	15
Asparagine (ASN)	2.42	69 ^Q,C^, 155 ^C^, 141 ^C^	75
Aspartic acid (ASP)	3.00	216 ^Q,C^, 130 ^C^, 88 ^C^	5
Methionine (MET)	3.03	101 ^Q,C^, 277 ^C^, 203 ^C^	5
Hydroxyproline (HYP)	3.17	172 ^Q,C^, 86 ^C^, 68 ^C^	15
Glutamic acid (GLU)	3.37	230 ^Q,C^, 170 ^C^, 84 ^C^	25
Phenylalanine (PHE)	3.40	148 ^Q,C^, 206 ^C^, 190 ^C^	3
Glutamine (GLN)	4.08	84 ^Q,C^, 187 ^C^, 112 ^C^	11
Lysine (LYS)	4.73	170 ^Q,C^, 153 ^C^, 128 ^C^	4
Histidine (HIS)	4.92	81 ^Q,C^, 282 ^C^, 168 ^C^	40
Tyrosine (TYR)	5.22	107 ^Q,C^, 206 ^C^, 164 ^C^	3
Tryptophan (TRP)	5.52	130 ^Q,C^, 332 ^C^, 229 ^C^	19

^Q^ Quantification ions; ^C^ Confirmation ions; IS, internal standard; NE, not evaluated.

**Table 2:** HPLC-MS data and limits of quantification for the studied free amino acids.

Amino Acid (Abbreviation)	Retention Time (min)	Ions (*m/z*)	LOQ (mg/kg)
Arginine (ARG)	3.09	303 ^Q,C^, 70 ^C^, 156 ^C^	270
Homoarginine (HARG; IS)	3.25	317 ^Q,C^, 128 ^C^, 84 ^C^	NE
Glutamine (GLN)	3.22	275 ^Q,C^, 172 ^C^, 84 ^C^	130
Serine (SER)	3.60	234 ^Q,C^, 174 ^C^, 146 ^C^	20
Asparagine (ASN)	3.72	243 ^Q,C^, 157 ^C^, 115 ^C^	210
Hydroxyproline (HYP)	3.90	260 ^Q,C^, 172 ^C^, 157 ^C^	160
Glycine (GLY)	4.10	204 ^Q,C^, 248 ^C^, 144 ^C^	40
Threonine (THR)	4.20	248 ^Q,C^, 188 ^C^, 160 ^C^	100
Alanine (ALA)	5.07	218 ^Q,C^, 158 ^C^, 130 ^C^	9
γ-Amino-n-butyric acid (GABA)	5.49	232 ^Q,C^, 172 ^C^, 130 ^C^	10
Sarcosine (SAR)	5.70	218 ^Q,C^, 158 ^C^, 88 ^C^	40
Ornithine (ORN)	6.50	347 ^Q,C^, 287 ^C^, 156 ^C^	15
Methionine-*d3* (MET-*d3*; IS)	6.80	281 ^Q,C^, 221 ^C^, 193 ^C^	NE
Methionine (MET)	6.88	278 ^Q,C^, 218 ^C^, 190 ^C^	50
Proline (PRO)	6.95	244 ^Q,C^, 184 ^C^, 156 ^C^	8
Lysine (LYS)	7.55	361 ^Q,C^, 301 ^C^, 170 ^C^	65
Aspartic acid (ASP)	7.57	304 ^Q,C^, 216 ^C^, 130 ^C^	35
Histidine (HIS)	7.60	370 ^Q,C^, 196 ^C^, 110 ^C^	17
Valine (VAL)	7.96	246 ^Q,C^, 158 ^C^, 116 ^C^	80
Glutamic acid (GLU)	8.06	318 ^Q,C^, 258 ^C^, 172 ^C^	16
Tryptophan (TRP)	8.46	333 ^Q,C^, 273 ^C^, 245 ^C^	20
Leucine (LEU)	9.50	260 ^Q,C^, 172 ^C^, 74 ^C^	16
Phenylalanine (PHE)	9.68	294 ^Q,C^, 206 ^C^, 120 ^C^	45
Isoleucine (ILE)	9.95	260 ^Q,C^, 172 ^C^, 74 ^C^	96
Tyrosine (TYR)	12.25	396 ^Q,C^, 308 ^C^, 136 ^C^	48

^Q^ Quantification ions; ^C^ Confirmation ions; IS, internal standard; NE, not evaluated.

**Table 3:** GC-MS and HPLC-MS conditions.

GC-MS Parameter	Final Setting
Programmed temperature conditions	from 110 °C to 320 °C (0 min), at 30 °C/min
Carrier gas	Helium
Flow-rate (mL/min)	1.1
Injector temperature	250
Injection volume (L)	2
Injection mode	Splitless
MS operating mode	Electron impact
Scan range (*m*/*z*)	50–450
MS temperatures	ion source 240 °C, quadrupole 180 °C, and auxiliary 310 °C
**HPLC-MS parameter**	**Final setting**
Gradient elution mode	Ammonium formate (10 mM) in water (A) and ammonium formate (10 mM) in methanol (B): (i) 0.00 min (A:B, 32:68, *v*/*v*); (ii) 13.00 min (A:B, 17:83, *v*/*v*); (iii) 13.01 min (A:B, 32:68, *v*/*v*); (iv) 17.00 min (A:B, 32:68, *v*/*v*)
Flow-rate (mL/min)	0.5
Injection volume ((L)	5
Temperature (°C)	35
MS Ionization source	ESI
Scan range (*m*/*z*)	60–600
Capillary voltage (V)	3500
Fragmentor voltage (V)	60
Drying gas (N2) flow (L/min)	8
Drying gas (N2) temperature (°C)	325
Nebulizer gas pressure (psi)	40

**Table 4:** Calibration curve data (*n* = 3).

Amino Acid	GC-MS			HPLC-MS		
	SCI (SS)	SCI (MMS)	ME *	SCI (SS)	SCI (MMS)	ME *
ALA	0.073–0.078	0.058–0.078	−11	0.005–0.008	0.020–0.047	80
ARG	NE	NE	NE	0.001–0.015	0.002–0.017	16
ASN	0.024–0.028	0.019–0.025	−18	0.001–0.003	0.001–0.003	4
ASP	0.039–0.044	0.042–0.045	5	0.023–0.032	0.021–0.032	4
GABA	0.003–0.004	0.002–0.004	−16	0.001–0.009	0.001–0.011	16
GLN	0.008–0.011	0.017–0.020	48	0.001–0.019	0.001–0.024	19
GLU	0.006–0.010	0.006–0.012	11	0.001–0.021	0.001–0.025	16
GLY	0.048–0.064	0.048–0.059	−4	0.006–0.011	0.012–0.036	64
HIS	0.006–0.007	0.007–0.010	23	0.071–0.087	0.061–0.069	−22
HYP	0.043–0.047	0.037–0.044	−11	0.001–0.004	0.001–0.005	16
ILE	0.016–0.023	0.011–0.014	−56	0.035–0.038	0.018–0.019	−97
LEU	0.059–0.073	0.034–0.041	−76	0.077–0.079	0.055–0.069	−26
LYS	0.010–0.023	0.024–0.026	34	0.001–0.024	0.041–0.046	71
MET	0.017–0.019	0.014–0.018	−12	0.027–0.040	0.024–0.027	−31
ORN	NE	NE	NE	0.002–0.022	0.053–0.093	84
PHE	0.006–0.007	0.019–0.026	71	0.025–0.036	0.029–0.037	9
PRO	0.063–0.068	0.012–0.043	−138	0.095–0.110	0.075–0.090	−24
SAR	NE	NE	NE	0.024–0.029	0.030–0.045	29
SER	0.015–0.020	0.014–0.016	−16	0.002–0.003	0.006–0.011	70
THR	0.033–0.039	0.033–0.036	−4	0.005–0.017	0.004–0.015	−15
TRP	0.081–0.090	0.086–0.111	12	0.003–0.018	0.038–0.052	77
TYR	0.005–0.006	0.045–0.062	88	0.003–0.020	0.011–0.015	−11
VAL	0.033–0.040	0.029–0.040	−6	0.085–0.145	0.025–0.033	−290

SCI, slope confidence interval; SS, standard in solvent; ME, matrix effect; MMS, matrix-matched standards; NE, not evaluated; * ME was calculated as follows: 100 × [1 − (SS slope/MMS slope)].

**Table 5:** Comparison of the GC-MS and HPLC-MS methods.

Amino Acid	GC-MS	HPLC-MS
Number of amino acids identified	20	23
Chromatographic run time (min)	7	21 *
Overall method time (min)	30	50 *
Limit of quantification range (mg/kg)	3–75	8–270
Number of amino acids with significant matrix effect	8	13

* Two injections should be done for HPLC-MS (+21 min) if the minority compounds were not observed in the diluted sample.

**Table 6:** Results (means of triplicate analyses (mg/kg; dry weight); %RSD < 15% in all cases) of the investigation of bee pollen samples from commercial origin (C1–C8).

Amino Acid	C1	C2	C3	C4	C5	C6	C7	C8
**ALA**	86	131	334	250	249	348	119	136
**GLY**	20	50	25	16	30	27	12	14
**VAL**	45	82	102	83	107	92	59	62
**LEU**	ND	77	74	52	61	72	4	8
**ILE**	28	58	90	67	90	86	38	41
**THR**	23	141	52	36	51	47	26	28
**GABA**	70	421	420	226	635	339	76	75
**SER**	75	2200	159	113	170	125	81	84
**PRO**	3446	2197	4187	3873	3499	4577	2937	3214
**ASN**	270	162	698	303	1042	403	483	471
**ASP**	178	115	281	219	281	239	273	200
**MET**	<LOQ	98	10	15	10	10	<LOQ	<LOQ
**HYP**	140	194	234	192	458	187	155	170
**GLU**	167	122	381	316	483	249	353	365
**PHE**	ND	48	44	18	28	42	ND	ND
**GLN**	23	92	55	31	39	58	26	26
**LYS**	28	117	75	47	94	70	30	32
**HIS**	ND	103	363	<LOQ	73	304	< ND	ND
**TYR**	4	92	28	28	62	29	4	10
**TRP**	78	152	173	91	71	1175	56	60
**TOTAL**	**4681**	**6652**	**7785**	**5976**	**7533**	**8479**	**4732**	**4996**

ND, not detected; <LOQ, below the limit of quantification.

**Table 7:** Results (means of triplicate analyses (mg/kg; dry weight); %RSD < 15% in all cases) of the investigation of bee pollen samples from experimental apiaries (A1–A4).

Amino Acid	A1	A2	A3	A4
ALA	370	454	314	241
GLY	64	69	39	44
VAL	307	208	95	193
LEU	215	175	22	38
ILE	643	223	65	125
THR	182	99	45	96
GABA	683	1190	805	2025
SER	648	268	235	471
PRO	974	2250	1917	2878
ASN	1201	643	2609	4913
ASP	446	279	833	454
MET	15	11	10	14
HYP	120	108	404	202
GLU	1985	744	365	405
PHE	27	19	168	28
GLN	217	179	99	412
LYS	302	221	245	205
HIS	228	258	1808	633
TYR	30	28	<LOQ	22
TRP	294	163	29	44
TOTAL	8951	7589	10,107	13,443

<LOQ, below the limit of quantification.

## Data Availability

The datasets generated during the current study are contained within this article and the Supplementary Materials, or they are available from the corresponding author on reasonable request.

## Supplementary Materials

The following supporting information can be downloaded at: https://www.mdpi.com/article/10.3390/foods11244013/s1, Figure S1: Location and Global Positioning System (GPS) data of the apiaries (Fuentelahiguera, FH; Pistacho, PI; Monte, MO; Tio Natalio, TN). Adapted from Foods, 11, Ares, A. M., Tapia, J. A., González-Porto, A. V., Higes, M., Martín-Hernández, R, Bernal, J., Glucosinolates as markers of the origin and harvesting period for discrimination of bee pollen by UPLC-MS/MS, 1446, 2022, with permission from MDPI; Figure S2: Results of the flow injection analysis of a GLN standard solution for optimizing the value of the fragmentor voltage (20–380 V; F20–F380); Figure S3: MS spectra of VAL in (A) solvent and (B) matrix-matched standards. The GC-MS conditions are summarized in Section 2.4.1; Figure S4: MS spectra of GLY in (A) solvent and (B) matrix-matched standards. The HPLC-MS conditions are summarized in Section 2.4.2; Table S1: Summary of trueness studies; Table S2: Weights of the five principal components.; Table S3: Eigenvalues of the correlation matrix; Table S4: Number of observations and percentage classified in each group using a quadratic discriminant analysis.
