# Supplementary material for: Fast Chromatographic Determination of Free Amino Acids in Bee Pollen
Source: Foods. 2022 Dec 12;11(24):4013. doi: 10.3390/foods11244013 (PMC9778440; doi:10.3390/foods11244013)
Supplement: Supplementary file 1 [file foods-11-04013-s001.zip › foods-2074927-supplementary.pdf]

Supplementary Material

Fast chromatographic determination of free amino acids in bee pollen

Beatriz Martín-Gómez [1], Laura Salahange [1], Jesús A. Tapia [1,2], María T. Martín [1], Ana M. Ares [1], and José Bernal [1,*]

[1] Analytical Chemistry Group (TESEA), I. U. CINQUIMA, Faculty of Sciences, University of Valladolid, 47011 Valladolid, Spain
[2] Department of Statistics and Operations Research, Faculty of Sciences, University of Valladolid, 47011 Valladolid, Spain
* Correspondence: jose.bernal@uva.es (J.B.); Tel.: +34-983186347; https://tesea.uva.es/

Table S1. Summary of trueness studies for some of the studied amino acids.

	GC-MS			HPLC-MS		
	Mean (%) ± RSD (%)			Mean (%) ± RSD (%)		
**Amino acid**	Low	Medium	High	Low	Medium	High
**ALA**	99 ± 8	103 ± 13	100 ± 7	95 ± 10	98 ± 7	102 ± 8
**GLU**	92 ± 12	95 ± 8	87 ± 10	90 ± 8	97 ± 14	95 ± 11
**LEU**	102 ± 8	97 ± 4	99 ± 7	98 ± 6	104 ± 4	99 ± 8
**PRO**	91 ± 7	94 ± 9	96 ± 6	94 ± 12	92 ± 3	95 ± 7
**TRP**	88 ± 11	86 ± 8	87 ± 5	92 ± 9	90 ± 3	93 ± 9
**VAL**	85 ± 5	88 ± 6	91 ± 2	90 ± 6	88 ± 10	85 ± 8

Low- LOQ (see Tables 1 and 2); Medium QC-50 nmol/mL; High QC-200 nmol/mL.

Table S2.. Weights of the five principal components.

	Prin1	Prin2	Prin3	Prin4	Prin5
**ALA**	0.236057	-0.036155	0.177358	0.311309	0.339895
**GLY**	0.314397	-0.031592	0.056109	0.055225	0.109943
**VAL**	0.303928	-0.100335	-0.177942	0.012101	0.143828
**LEU**	0.262050	-0.263823	0.072527	0.183980	0.030612
**ILE**	0.275516	-0.206456	-0.137586	0.141888	-0.122005
**THR**	0.309164	-0.167850	-0.006714	-0.040024	-0.074947
**GABA**	0.234345	0.165024	0.029122	-0.362413	0.311457
**SER**	0.214260	-0.101585	0.284065	-0.252636	-0.336496
**PRO**	-0.251365	-0.064490	0.285289	-0.064884	0.361294
**ASN**	0.169649	0.311969	-0.063674	-0.374812	0.189562
**ASP**	0.184648	0.388299	-0.047240	0.169538	-0.101457
**MET**	0.134758	-0.079638	0.511498	-0.185445	-0.239149
**HYP**	-0.019473	0.314684	0.288880	0.132603	-0.100750
**GLU**	0.261909	-0.186448	-0.199916	0.174555	-0.168466
**PHE**	0.099522	0.383165	0.228584	0.298679	-0.107669
**GLN**	0.254724	0.078593	-0.089529	-0.401106	0.242718
**LYS**	0.322003	0.106982	-0.038615	0.068153	-0.031016
**HIS**	0.129948	0.428577	0.080410	0.175902	-0.005519
**TYR**	0.059226	-0.220286	0.500309	-0.098823	-0.013847
**TRP**	0.002956	-0.143675	0.191574	0.312750	0.525214

Table S3. Eigenvalues of the correlation matrix.

Principal component	Eigenvalue	Difference	Proportion	Accumulated variability (%)
**1**	8.99183530	4.9626684	0.4496	44.96
**2**	4.0291669	1.7987107	0.2015	65.11
**3**	2.2304562	0.4620516	0.1115	76.26
**4**	1.7684046	0.4078791	0.0884	85.10
**5**	1.3605255		0.0680	91.90

Table S4. Number of observations and percentage classified in each group using a quadratic discriminant analysis.

Origin	AP	COM	Total
**AP**	4	0	7
%	100.00	0.00	100.00
**COM**	0	8	8
%	0.00	100.00	100.00
**Total**	4	8	12
	33.33	66.67	100.00

AP, samples from experimental apiaries; **COM**, commercial samples.

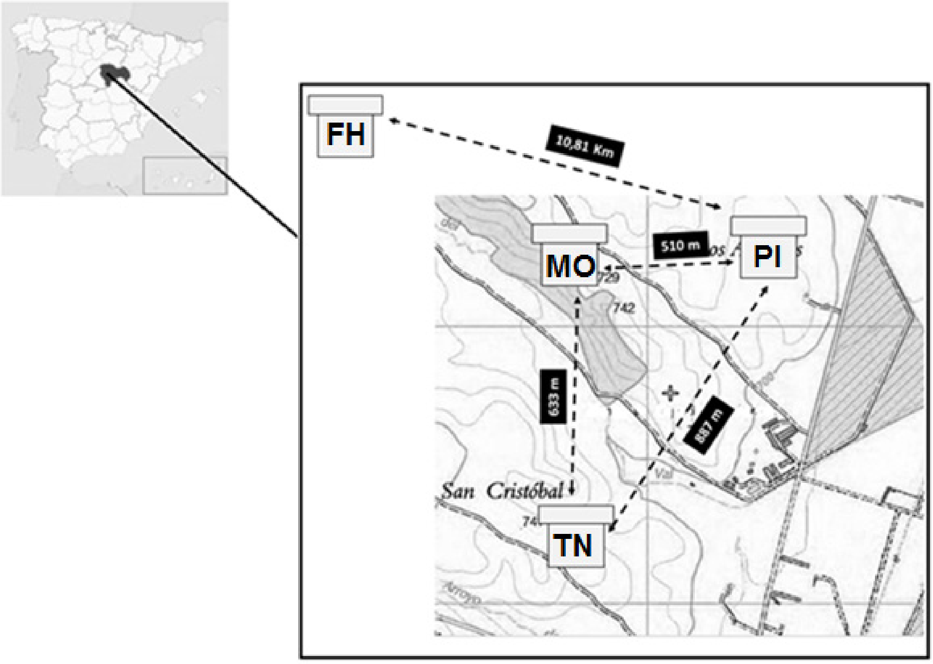

	X	Y
TN	482.405.65	4.503.154.12
PI	482.370.46	4.503.607.58
MO	482.255.70	4.503.388.39
FH	474.340.88	4.511.226.24

Figure S1.- Location and Global Positioning System (GPS) data of the apiaries (Fuentelahiguera, FH; Pistacho, PI; Monte, MO; Tio Natalio, TN). Adapted from Foods, 11, Ares, A. M., Tapia, J. A., González-Porto, A. V., Higes, M., Martín-Hernández, R, Bernal, J., Glucosinolates as markers of the origin and harvesting period for discrimination of bee pollen by UPLC-MS/MS, 1446, 2022, with permission from MDPI.

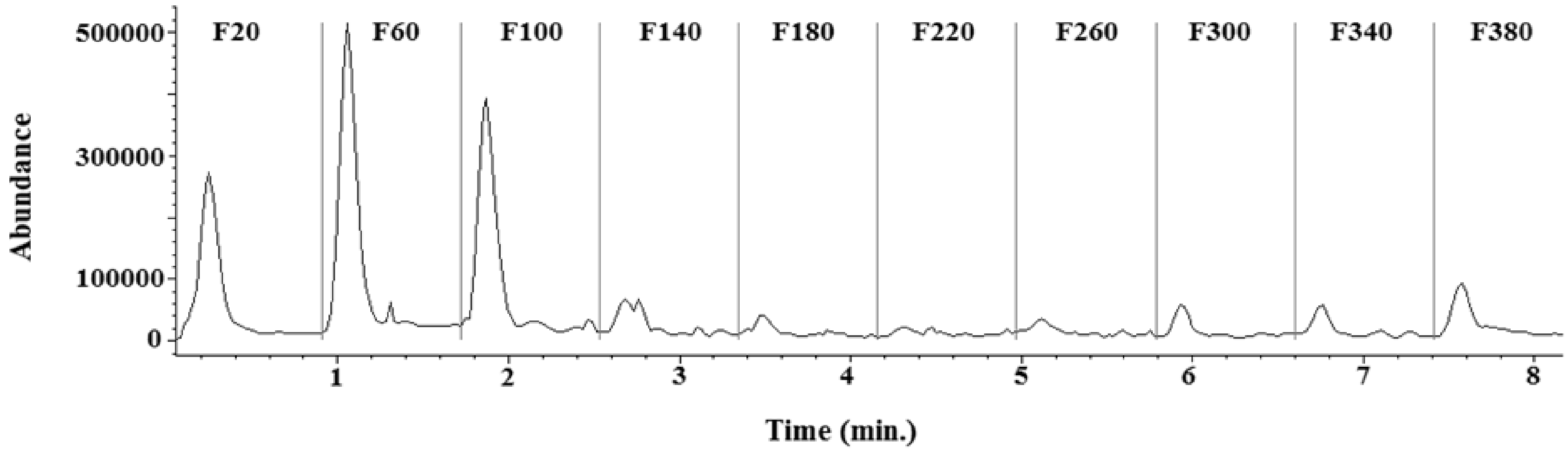

Figure S2. Results of the flow injection analysis of a GLN standard solution for optimizing the value of the fragmentor voltage (20-380V; F20-F380).

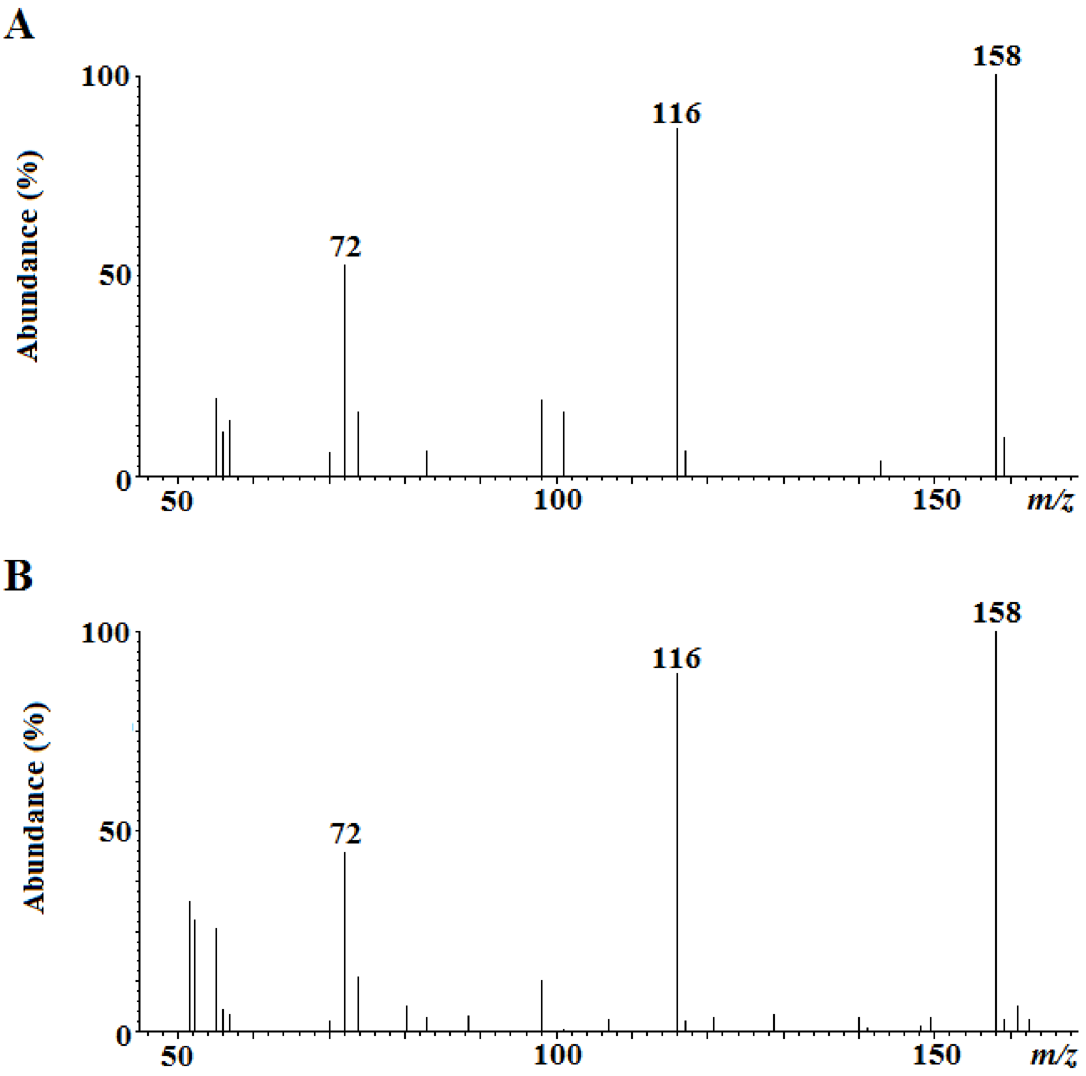

Figure S3. MS spectra of VAL in (A) solvent and (B) matrix-matched standards. The GC-MS conditions are summarized in subsection 2.4.1.

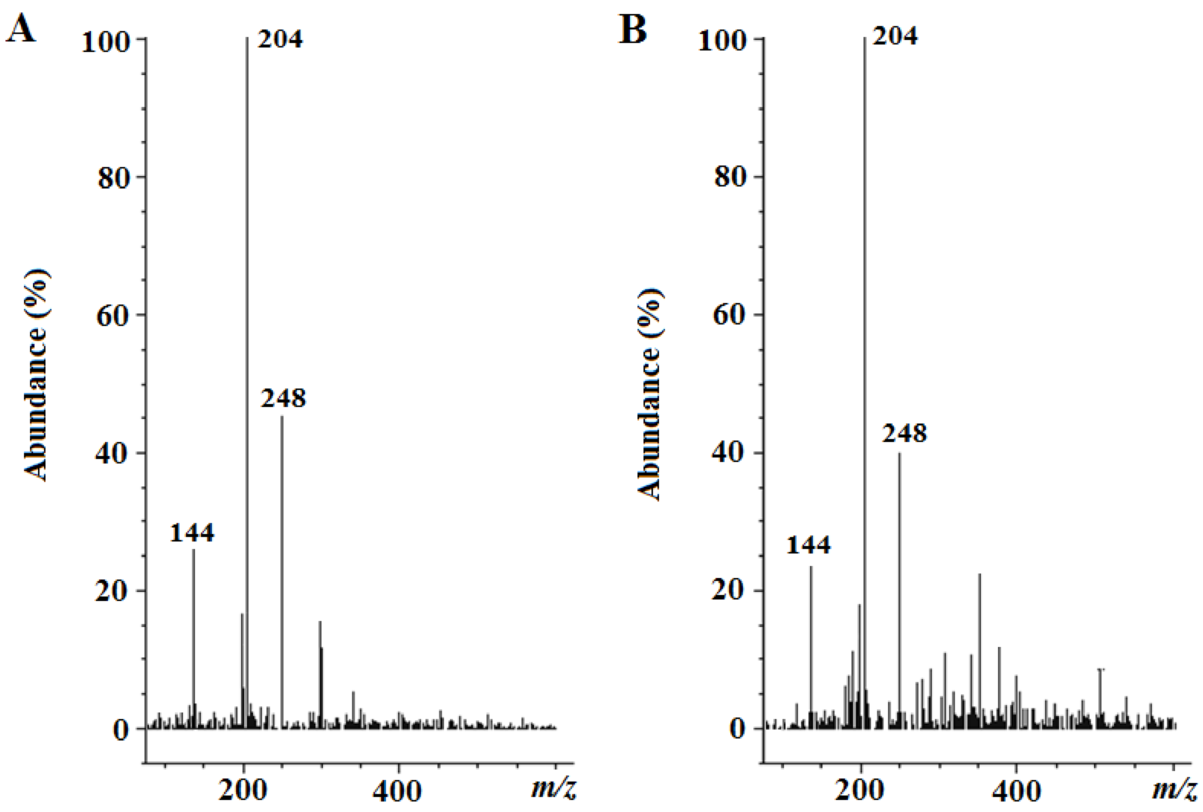

Figure S4. MS spectra of GLY in (A) solvent and (B) matrix-matched standards. The HPLC-MS conditions are summarized in subsection 2.4.2.
